# Clinical Characterization of Pathogens, Risk Factors and Quality of Life in an Observational Study of Native vs. Prosthetic Aortic Valve Endocarditis Surgery

**DOI:** 10.3390/life14081029

**Published:** 2024-08-19

**Authors:** Anton Heller, Matthäus Zerdzitzki, Philipp Hegner, Zhiyang Song, Christian Schach, Florian Hitzenbichler, Kostiantyn Kozakov, Claudius Thiedemann, Zdenek Provaznik, Christof Schmid, Jing Li

**Affiliations:** 1 Department of Cardiothoracic Surgery, University Medical Center Regensburg, Franz-Josef-Strauss-Allee 11, 93053 Regensburg, Germanykostiantyn.kozakov@ukr.de (K.K.); zdenek.provaznik@ukr.de (Z.P.); christof.schmid@ukr.de (C.S.); jing.li@ukr.de (J.L.); 2 Department of Vascular Surgery, University Medical Center Regensburg, 93053 Regensburg, Germany; 3 Department of Internal Medicine II—Cardiology, University Medical Center Regensburg, 93053 Regensburg, Germany; philipp.hegner@ukr.de (P.H.); christian.schach@ukr.de (C.S.); 4 Institute of Mathematics, Ludwig-Maximilian University Munich, 80539 Munich, Germany; 5 Department of Infectiology, University Medical Center Regensburg, 93053 Regensburg, Germany; florian.hitzenbichler@ukr.de; 6 Department of Orthopedics and Trauma Surgery, University Medical Center Regensburg, 93053 Regensburg, Germany; claudius.thiedemann@ukr.de; 7 Department of Occupational Medicine, University Medical Center Regensburg, 93053 Regensburg, Germany

**Keywords:** aortic valve endocarditis, native valve endocarditis, prosthetic valve endocarditis, health-related quality of life, Minnesota Living with HF Questionnaire

## Abstract

**Background:** Native (NVE) and prosthetic (PVE) aortic valve endocarditis (AVE) remain a surgical challenge with an ongoing trend towards more complex surgical procedures. **Methods:** First-time NVE was compared with PVE, focusing on pathogens, risk factors, perioperative course, postoperative follow-up, including recurrent infection, as well as health-related quality of life (HRQOL). **Results:** From 2007 to 2022, surgical intervention for AVE was necessary in 231 patients with 233 episodes of infective aortic valve endocarditis, i.e., there were only two cases of reinfection (NVE group). The study group consisted of 130 cases with NVE and 103 with PVE. Overall, a median of 40.3% of survivors were in NYHA class I or II. In-hospital mortality was higher in the PVE group with 13.3%. The most common pathogen was *Staphylococcus aureus*, with 24.9% across both groups. EuroSCORE II was higher in the PVE group (19.0 ± 14.3% total, NVE 11.1 ± 8.1%, PVE 27.8 ± 14.6%; *p* < 0.05), reflecting an older, more co-morbid patient cohort. Abscess formation was also more common in the PVE group, while vegetations were more common in the NVE group. The 5-year and 10-year survival rates did not differ significantly between NVE and PVE and were 74.4% and 52.2% for the NVE group, respectively, and 67.4% and 52.9% for the PVE group, respectively. The HRQOL as assessed by the Minnesota Living with HF Questionnaire (MLHFQ) demonstrated no significant difference between both groups. **Conclusions:** Long-term survival and QoL after surgical treatment of infective aortic valve endocarditis are excellent and do not depend on the type of replacement.

## 1. Introduction

Despite its initial characterization by Horder in the mid-sixteenth century, infective endocarditis (IE) remains to be a disease with elusive pathophysiology, accompanied by an ongoing increase in morbidity and mortality [1,2]. A significant epidemiological shift has occurred within the clinical spectrum of aortic valve pathology. The primary underlying cause of aortic valve deterioration has shifted from rheumatic heart disease to senile and calcifying degenerative valve disease, as well as healthcare-associated infections of prosthetic valves [3]. Epidemiological data indicate that infective endocarditis has a higher incidence in industrialized nations, with 40% to 67% of cases affecting the aortic valve [4]. The surgical treatment of endocarditis remains challenging, especially in patients with prosthetic endocarditis. As the duration of heart ischemia and operation time increases, postoperative complications and mortality also increase accordingly [5].

Despite detailed expert guidelines on the presentation and management of infective endocarditis (IE), the in-hospital mortality for this condition remains 20–30%, a figure that has largely unchanged since the introduction of surgical treatment in the 1960s [6]. This persistent mortality rate is attributable to severe complications such as cerebrovascular embolic events, progressive heart failure, and irreversible structural damage from uncontrolled valvular infection [7].

Native aortic valve endocarditis can be either community acquired or healthcare related. In developing countries, rheumatic heart disease remains the primary risk factor. In developed countries, the predominant risk factors include degenerative valve disease, malignancy, intravenous drug use, diabetes mellitus, and congenital heart disease, with the mean patient age over 70 [8].

The pathophysiology of infective endocarditis involves bacteremia (either spontaneous or hospital acquired) delivering the pathogen to the valve surface, pathogen adherence to the prepared valve surface, and eventual invasion of the valvular leaflets. Circulating pathogens do not adhere to normal endothelium; however, valve injury alters endothelial cell architecture, making it susceptible to bacterial colonization [9].

Prosthetic valve endocarditis (PVE) is a microbial infection affecting the endovascular surfaces of a prosthetic valve or the reconstructed native valve, whereas native aortic valve endocarditis involves infection of the native valve without prior surgical reconstruction or replacement [10]. PVE accounts for approximately 20% of all endocarditis cases and is associated with high morbidity and mortality [11,12]. Early PVE is typically caused by *Staphylococcus aureus* and *Coagulase-negative staphylococci*, often due to cardiac structure injury during initial implantation. Late PVE, occurring beyond 12 months post-implantation, results from alterations in the valve and paravalvular surface, leading to microthrombi formation that facilitates bacterial adherence and infection [4].The modified Duke criteria, supplemented by imaging studies such as 18F-FDG PET/CT, are used in the diagnostic workup [13]. Key predictors of mortality include persistent bacteremia, heart failure, intracardiac abscess, and stroke [12]. Surgical intervention is required for curative therapy in up to 50% of hospitalized patients with IE [12,14].

The risk associated with surgical interventions for infective endocarditis correlates with multiple variables: the urgency of surgery, the extent of structural cardiac damage, and the presence of several preoperative risk factors, including advanced age and chronic renal failure [12,14]. While numerous studies have focused on short-term survival, long-term outcome remains less thoroughly investigated. There is a paucity of data regarding functional recovery (NYHA) and quality of life among survivors. Both of which are becoming increasing importance in an aging and increasingly morbid society.

In this study, we evaluated various aspects of patients with native and prosthetic aortic valve endocarditis, including clinical characteristics, surgical risk factors, operational data, detected pathogens, quality of life, in-hospital mortality, and long-term survival. Despite the fact that affected valve is the same in both cases, there are substantial difference between these patients’ populations. Additionally, we assessed quality of life using the widely established questionnaire (MLHFQ).

## 2. Materials and Methods

### 2.1. Patient Selection

This retrospective investigation targeted patients diagnosed with infective AVE who underwent cardiac surgery between January 2007 and December 2022 at the University Medical Center Regensburg, Germany. The University Medical Center Regensburg is an academic reference hospital institution and a maximum-care facility with over 800 beds, serving a population of more than 2 million people. Cases with concomitant mitral- or tricuspid valve endocarditis as well as multi-valve replacement were not included, as the primary focus of this study was aortic valve endocarditis.

### 2.2. Definitions

In this study, all-cause mortality variables were subdivided into in-hospital mortality, referring to deaths occurring prior to hospital discharge, and follow-up mortality, which encompasses deaths occurring post-discharge. The recurrence of endocarditis was characterized by the necessity for a subsequent cardiac surgical intervention due to valve reinfection. The definition of postoperative major stroke included the occurrence of any new cerebral infarction, which was confirmed through neurological evaluation and cerebral computed tomography imaging. Healthcare-associated endocarditis was delineated as endocarditis manifesting within a hospital setting (nosocomial) or as endocarditis arising extramurally in individuals with significant healthcare exposure, such as those frequenting day-care hospitals, dialysis centers, or outpatient parenteral antibiotic therapy programs, as well as residents in nursing facilities [8].

Assessment of the current health-related quality of life (HRQOL) was conducted utilizing the Minnesota Living with Heart Failure Questionnaire (MLHFQ), a validated patient self-reporting questionnaire. This disease-specific questionnaire encompasses 21 items, each quantified via a six-point Likert scale to gauge the impact of heart failure on the patient’s quality of life. Scores range from 0 (no impact) to 5 (maximum impact), yielding a cumulative score between 0 (optimal HRQOL) and 105 (poorest HRQOL). The MLHFQ further defines a physical dimension (8 items: 2, 3, 4, 5, 6, 7, 12, 13; score range 0–40) and an emotional dimension (5 items: 17, 18, 19, 20, 21; score range 0–25). The remaining eight items contribute solely to the total score, with higher values indicating a more severe HRQOL impairment [15].

Renal failure was classified using RIFLE criteria. For this study, postoperative renal failure was classified as temporary or permanent regarding the need for hemodialysis [16].

Treatment was performed in accordance with national and international guidelines according to the standard of care at the clinician’s discretion. Vegetations were typically assessed by transesophageal echocardiography (Table 1). Embolism was typically assessed by computed tomography (CT), magnetic resonance imaging, and/or nuclear imaging. The search for the pathogen in cases of suspected endocarditis was conducted at the discretion of the attending clinician. Blood cultures were processed in the facility’s department of microbiology, where they were cultured, and the microorganism was determined.

### 2.3. Indication for Surgery

Surgical intervention was indicated in cases of pronounced valvular dysfunction combined with deterioration of ventricular function (evidenced by hemodynamic instability necessitating vasopressor or inotropic support), isolation of antibiotic-resistant pathogens, ongoing infection or persistent bacteremia, as well as the presence of sizable mobile vegetations, repeated embolic events, or the development of paravalvular abscesses, in accordance with the modified Duke criteria for the diagnosis of endocarditis [12].

### 2.4. Surgical Approach

Surgical management of AVE typically involved a median sternotomy approach, with induction of cardiac arrest and maintenance of circulation via a conventional cardiopulmonary bypass. The operative protocol mandated meticulous debridement of all infected material and the extensive evacuation of accessible abscess cavities. Reconstruction frequently involved sealing abscess cavities and necrotic fistulas with bovine pericardial patches, thereby restoring the structural integrity of the aortic annulus. Prosthetic aortic valve replacement was performed in accordance with established surgical standards. Perioperative antimicrobial therapy was administered in compliance with the EACTS/ESC European guidelines, extending for a minimum duration of six weeks [12].

### 2.5. Statistical Analysis

Data were systematically extracted utilizing SAP (ERP 6.0, Walldorf, Germany) and Swisslab (Roche Diagnostics IT Solutions, Berlin, Nexus AG, Berlin, Germany), subsequently compiled into a Microsoft Excel spreadsheet (Microsoft Excel 2019, Redmond, WA, USA) in a pseudonymized format. Categorical variables were delineated by absolute and relative frequencies. Depending on the distribution, continuous variables were represented as means with standard deviations (SD) or medians accompanied by minimum-maximum ranges, contingent upon their distributional characteristics. Group comparisons were performed using the unpaired Student’s t-test or Mann–Whitney U-test. Discrete variables were expressed as percentages and tested with the chi-squared test or Fisher’s exact test. For the distribution of health-related quality of life (HRQOL) scores, one-way ANOVA tests were utilized to discern group differences. Long-term survival rates were depicted through Kaplan–Meier survival curves. Logistic regression models were employed to calculate independent odds ratios for variables associated with in-hospital mortality. Missing data were not imputed and were randomly assumed to be missing. Statistical significance was assumed with *p*-values undercutting 0.05.

### 2.6. Study Approval

Ethical approval for this study was granted by the Institutional Review Board of the University of Regensburg, Germany (approval number 20-1912-104). All research was conducted in accordance with the Declaration of Helsinki (most recent revision in 2013). Written informed consent was waived based on the retrospective nature of the study.

## 3. Results

### 3.1. Baseline Patient Characteristics

NVE was prevalent in 130 (55.8%) patients, while PVE accounted for 103 (44.2%) cases (Table 1). The median age for patients with PVE was 69.8 years, compared to 62.0 years for those with NVE (*p* < 0.05). Coronary artery disease was present in 23.1% of NVE patients and 49.5% of PVE patients (34.8% in total, *p* < 0.05), with no additional cases diagnosed intraoperatively. A severely reduced left-ventricular ejection fraction (<30%) was observed at a mean of 4.3% in the total patient cohort, 0.8% of NVE, and 8.7% of PVE (*p* < 0.05). Additionally, median logistic EuroSCORE II was 19.0 ± 14.3% in total, 11.1 ± 8.1% for NVE, and 27.8 ± 14.6% for PVE (*p* < 0.05). Similarly, preoperative inflammatory markers were found to be comparable in both cohorts (Table 2). In the PVE cohort, 40 cases (38.8%) presented as early endocarditis, and 63 (61.2%) as late endocarditis. Also, of 103 total PVE cases, 40 had a biological valve previously, whereas a mechanical valve was infected in 63 cases. Over the study period, only two cases of reinfection were observed.

### 3.2. Pathogens

The probable portal of entry was identified in 62.2% of the patients, as determined by the attending clinicians during treatment. Health care-associated aortic valve endocarditis was notably prevalent. Dental procedures accounted for 7.7% of infections (NVE 6.2% vs. PVE 9.7%, *p* = 0.44) (Table 3). 12.0% of cases were traced back to gastrointestinal interventions (NVE 13.8% vs. PVE 9.7%, *p* = 0.45), including colonoscopy, diverticular disease, colorectal carcinoma and adenoma resections, gastric surgeries, cholecystectomy, herniorrhaphy, and appendectomy. Bronchopulmonary infections led to endocarditis in 3.9% of cases (NVE 4.6% vs. PVE 2.9%, *p* = 0.73), while the genitourinary tract was implicated in only 1.3% of instances (NVE 2.3% vs. PVE 0%, *p* = 0.26). 7.3% of individuals with endocarditis presented with chronic kidney disease on hemodialysis (NVE 6.9% vs. PVE 7.8%, *p* > 0.99) or with port infections. Trauma surgery, predominantly after vehicular accidents, was associated with 6.9% of endocarditis cases (NVE 8.5% vs. PVE 4.9%, *p* = 0.41). Vascular surgery and cardiac catheterizations were identified as the entry point in 12.0% of patients (NVE 13.8% vs. PVE 9.7%, *p* = 0.41). Among non-healthcare-associated cases, intravenous drug use accounted for 1.7% (no significant difference between both groups), while meningitis and spondylodiscitis were responsible for 10.7% (NVE 0.8% vs. PVE 22.3%, *p* < 0.05) and 5.2% (NVE 2.3% vs. PVE 8.7%, *p* = 0.06) of infections, respectively, with a higher prevalence in the PVE group. In 37.8% of the patients, the mechanism of entry remained elusive.

**Figure 1:**
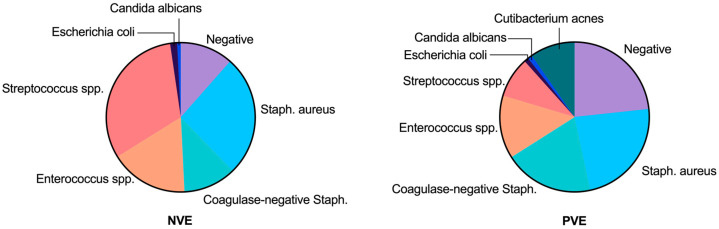
Causal pathogens in native (NVE) vs. prosthetic aortic valve endocarditis (PVE) cases. Negative indicates sterile blood cultures.

*Staphylococcus* species were the predominant pathogens, representing 39.9% of infections, with *Staphylococcus aureus* alone responsible for 24.9% of total infections (Table 4). The distribution across NVE and PVE cases is depicted in Figure 1. *Staphylococcus aureus* was the most frequently isolated pathogen across all groups. In our study, *Coagulase-negative staphylococci* were identified as the second most common etiological agents, representing a significant proportion of infections in both the overall sample and in cases of prosthetic valve endocarditis (PVE). *Candida albicans* yeast infections were confirmed in only two cases (Table 4).

### 3.3. Procedural Data

The duration of surgery, including cardiopulmonary bypass time, was 95.9 ± 44.0 min for NVE and 125.3 ± 56.9 min for PVE (*p* < 0.05), the aortic cross-clamp time was 83.2 ± 33.7 min for NVE and 113.1 ± 58.4 min for the PVE cohort (*p* < 0.05), thus showing significantly prolonged procedures for the PVE group. There was also a notable difference in valve replacement type: mechanical valve substitutes were more frequently utilized in the NVE group (NVE 26.2% vs. PVE 14.6%; *p* < 0.05), while biological valve replacement more commonly used in the PVE group (NVE 73.8% vs. PVE 85.4%; *p* < 0.05, Table 5). Vegetations were significantly more prevalent in the NVE group (81.5% versus 52.4% for PVE; *p* < 0.05), while as abscess formation was significantly more common in the PVE group (56.3% compared to 26.9% for NVE; *p* < 0.05) (Table 1).

### 3.4. In-Hospital Mortality

The in-hospital mortality rate was 13.3%, including 11 patients with NVE and 19 patients with PVE, the mortality rate was significant higher in the PVE group (NVE: 8.5% vs. PVE: 18.4%, *p* < 0.05) (Table 6). The primary causes of death were multi-organ failure and sepsis, which accounted for 19 fatalities (NVE: 8, PVE: 11) due to the uncontrolled infectious process. Additionally, three patients with PVE succumbed to intracranial hemorrhage. Respiratory failure in conjunction with pneumonia was the cause of death for one patient in each group (NVE vs. PVE). Others causes of death included acute mesenteric ischemia and aortic bleeding secondary to patch dehiscence.

Pathogen analysis in cases of in-hospital mortality identified staphylococcal infections as the predominant contributor, accounting for 64.5% of cases (with *Staphylococcus aureus* responsible for 48.4%), this was followed by streptococcal infections at 22.6%, *Enterococcus faecalis* at 6.5%, and *Candida albicans* infections at 3.2%.

In the majority of cases, in-hospital deaths were attributed to healthcare-associated infections accounting for 64% of fatalities. Further investigation revealed that post-vascular procedures associated with 13% of death, and chronic hemodialysis contributed to approximately 20%. Prior gastrointestinal surgical interventions were implicated in 13% of mortalities, while trauma requiring surgery was responsible for 6.5%.

### 3.5. Postoperative Morbidity

Postoperative complications were prevalent, with delirium occurring in 18.7% of cases, showing no significant difference between NVE and PVE (NVE: 17.8% vs. PVE: 19.8%, *p* = 0.72). Hemorrhage requiring re-thoracotomy was reported in 13.3% of patients and was significantly more common in the PVE group (NVE: 8.5% vs. PVE: 19.4%, *p* < 0.05). The need for secondary pacemaker insertion was noted in 12.9% of cases, with a higher incidence in the PVE group (NVE: 7.7% vs. PVE: 19.4%, *p* < 0.05). Extracorporeal life support (ECLS) was needed for low cardiac output syndrome in 4.7% of patients, with no significant difference between groups (NVE: 4.6% vs. PVE: 4.9%, *p* > 0.99).

New onset of postoperative stroke occurred in 4.7% of patients, with no significant difference between NVE and PVE groups (NVE: 5.4% vs. PVE: 3.9%, *p* = 0.76). Acute kidney injury requiring transient hemodialysis were observed in 12.9% of patients, with a significantly higher incidence in the PVE group (NVE: 7.7% vs. PVE: 19.4%, *p* < 0.05). The average duration of stay in the intensive care unit was 6.8 ± 10.1 days and in-hospital 18.8 ± 17.2 days. PVE patients experienced longer stays compared to NVE patients (ICU: NVE: 5.8 ± 6.4 days vs. PVE: 8.0 ± 13.2 days, *p* = 0.16), while the total duration of in-hospital stay being significantly longer in PVE patients (Hospital: NVE: 16.1 ± 11.0 days vs. PVE: 22.0 ± 22.1 days, *p* < 0.05) (Table 6)

### 3.6. Long Term Survival and QOL

**Figure 2:**
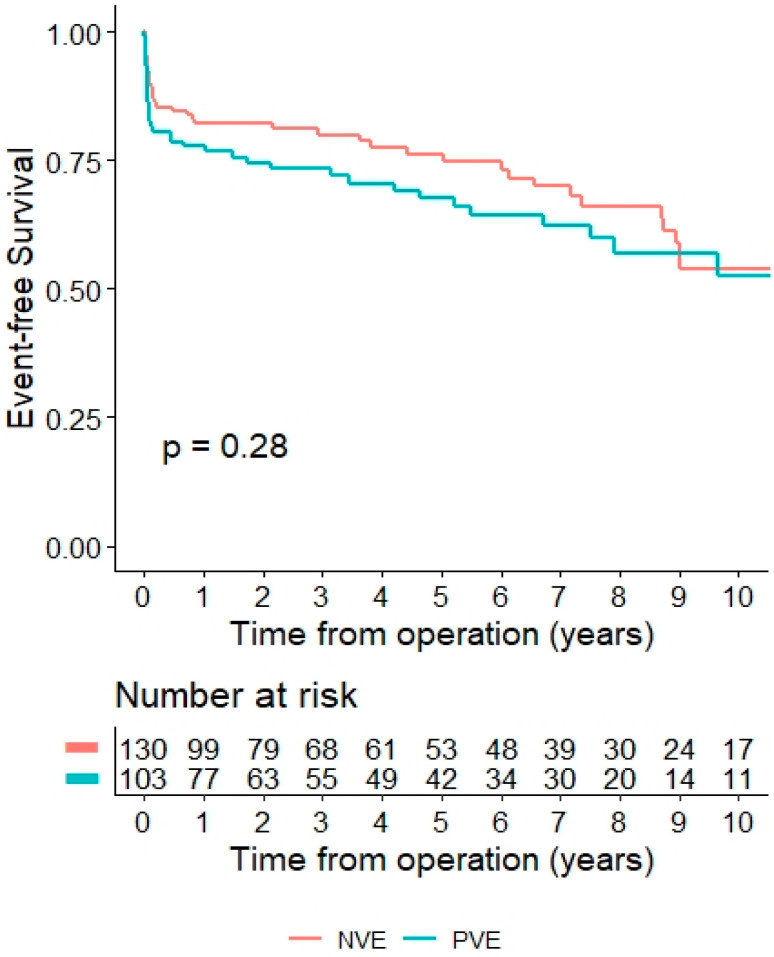
Postoperative survival and follow-up data, Kaplan–Meier survival estimates and patients at risk (NVE vs. PVE).

The mean follow-up duration in this study was 5.1 ± 4.0 years, during which 51 deaths occurred (NVE: 31 vs. PVE: 20, *p* = 0.50). The 5-year and 10-year survival rates were 74.4% and 52.2% for the NVE group, and 67.4% and 52.9% for the PVE group, respectively (Figure 2). An analysis of freedom from reinfection at the 14-year mark revealed rates of 98% for the NVE group and 100% for the PVE group, with two reinfections caused by MRSA in the NVE group.

**Figure 3:**
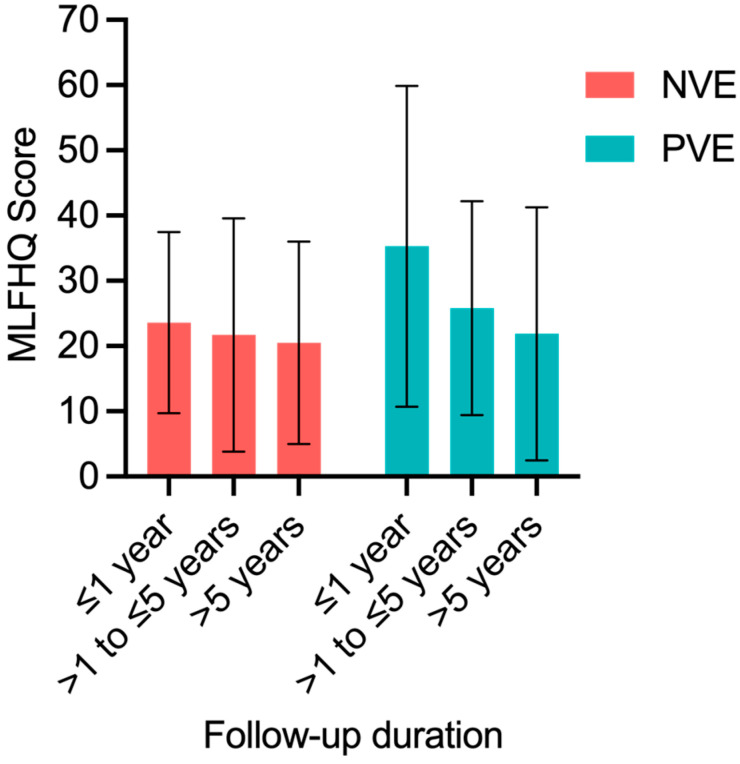
Quality of life measured with MLHFQ score in a long-term follow-up of patients with native aortic valve endocarditis (NVE) (*n* = 8 patients for ≤1 year, *n* = 25 for >1 to ≤5 years, *n* = 19 for >5 years) and prosthetic aortic valve endocarditis (PVE) (*n* = 3 patients for ≤1 year, *n* = 9 for >1 to ≤5 years, *n* = 15 for >5 years). Values represent mean ± SD. *p* = n.s. for NVE vs. PVE at all timepoints.

Out of both cohorts, 79 patients participated in the QOL assessment. The mean scores for the total MLHFQ were 26.5 ± 16.7 for a duration up to 1 year after surgery, 22.8 ± 17.4 for 1 to 5 years, and 21.1 ± 17.1 for over 5 years in the NVE group, with 23.6 ± 13.9, 21.7 ± 17.9, and 20.5 ± 15.5 in the PVE group, respectively, which did not differ significantly at all timepoints between groups (Figure 3).

### 3.7. Risk Factors

Multivariate logistic regression analysis identified several independent predictors of increased in-hospital mortality. Longer aortic cross-clamp time, chronic hemodialysis, infection with *Staphylococcus aureus*, and poor left ventricular ejection were strong predictors for in-hospital mortality (Table 7).

## 4. Discussion

Recent ESC data and other studies report mortality rate of 10–20% for NVE and 20–40% for PVE [12]. In our study, in-hospital mortality was low than in most previous reports. These wide ranges in operative mortality rates often reflect the varying clinical conditions of endocarditis patients at the time of surgery, particularly regarding the isolated pathogen and the presence of annular abscess formation. Previous reports on surgically treated patients with IE indicate that an annular abscess was present in 11–28% of the cases of native valve endocarditis, as well as in 40–63% of patients with prosthetic valve endocarditis [11,17].

In our study, annular abscesses were present in 26.9% of patients with native and 56.3% of those with prosthetic aortic valve endocarditis, which is consistent with current literature.

Patients with PVE experienced higher in-hospital mortality and more postoperative complications. They were generally older, had more comorbidities, and more frequently required complex cardiac redo surgery more often. Furthermore, PVE patients often needed additional procedures such as aortic surgery, reconstruction of the left ventricular outflow tract (LVOT) with pericardial patch plastic, or VSD closure [18]. During the study period, no significant changes in the materials or techniques used in PVE surgeries were observed.

Additionally, the incidence of coronary artery disease is associated with advanced age, given that the PVE cohort was older, a reduced left ventricular ejection fraction (LVEF) and a higher prevalence of a coronary artery disease were anticipated in this demographic [11]. Clinical signs of heart failure are also deemed to be rather unfavorable for the outcome following AVR [7].

The incidence of vegetations was significantly higher in the NVE group, potentially due to a superior adhesion capacity on native endothelial surfaces [9]. Conversely, abscess cavities were more frequently observed in cardiac tissue that had been previously subjected to surgical intervention, with a significantly higher predominance in patients with PVE. This finding aligns with existing literature. [17].

Our study highlights that infections, malignancies, or other lesions in the alimentary, genitourinary, and respiratory tracts to be much more firmly established than cutaneous lesions or injuries. Intravenous drug abuse, fractures, pregnancy and parturition, and any procedure involving the blood stream, particularly hemodialysis or cardiac catheterization, seemed to pose other possible foci of infection. This underscores the growing importance of the health care setting in relation to complications such as IE, particularly in an aging society that relies upon increasingly invasive and extensive medical care.

Analysis of the pathogen spectrum in our cohort revealed that *Staphylococcus aureus* posed the most problematic cause of infective endocarditis in general, especially in PVE [6,19]. Patients with PVE caused by *S. aureus* had the lowest overall survival. Several reports clearly show that these cases are best treated with the application of early cardiac surgery [5,19].

In our patient cohort, the prevalence of *Staphylococcus aureus* infection was 24.9%, corresponding well with the findings of other publications, with specifications going up to 26% prevalence of *Staphylococcus aureus* infections [19]. In addition to that, *Staphylococcus aureus* appeared to be the most aggressive pathogen, accounting for 48.4% of all in-hospital mortality.

The long-term survival of patients surgically treated for AVE at our center was quite satisfactory. Recurrence of infective endocarditis was rare, with a freedom from reinfection was 99% at 10 years, which is extraordinarily high as compared to existing literature [12,20]. The cases of recurrent endocarditis occurred in patients with MRSA as the predominant pathogen, and both showed paravalvular abscess formation at the time of the initial surgery. The reasons for the recurrent infections remain unclear. We hypothesize that inadequate debridement of all infected tissues or recurrent contamination of the prosthetic valve from an unidentified source may be responsible.

Many patients report persistent physically weakness and mentally imbalanced for a long time after surgery. Thus, simply description of survival and complications is insufficient to assess the long-term effects of surgical procedures. Instruments to measure QoL have been developed and are increasing used. To our knowledge, this is the first report of heart failure screening and patient related QoL after healed infective endocarditis, and there are no comparable data in the literature. In our study, the HF incidence after AVR was moderate at 14 years. The mean values of the physical and emotional MLHFQ scores of our patients were comparable with matched healthy populations and without significant disparities between the NVE and PVE groups. This observation may be confounded due to the fact that not all patients participated in the QoL questionnaire; for example, some patients died before hospital discharge.

## 5. Study Limitations

This investigation represents a single center experience with the surgical treatment of aortic valve endocarditis. The diagnosis of infective endocarditis mainly represents a challenge due to the frequent indeterminate origin of the infection and the nonspecific nature of its clinical manifestations. Retrospective analysis of pathogenic sources in this context was notably deficient. Optimal patient assessment would entail pre- and post-surgical evaluations to facilitate intraindividual comparisons. However, the urgency of the conditions and the critical preoperative states of the patients precluded comprehensive preoperative examinations. Additionally, the extended duration of the observation period introduced comorbid conditions such as cerebrovascular incidents and cardiac failure as potential confounding variables. The limited sample size of this study precludes any meaningful subgroup analysis. TAVI endocarditis and pacemaker-associated endocarditis were not the focus of the present study but will be the subject of future investigations.

## 6. Conclusions

This study highlighted the differences in treating native vs. prosthetic aortic valve endocarditis. PVE patients exhibited more comorbidities, resulting in a higher logistic EuroSCORE II, as well as longer extracorporeal circulation and cross-clamp times. Despite many similarities in the portal of entry and causative microorganisms between NVE and PVE. Our findings suggest that long-term survival and QoL after surgical treatment of infective aortic valve endocarditis do not depend on the type of replacement, despite the PVE group indicating a more severe acute phase. Notably, in-hospital mortality rates over 10% highlight the severe impact of endocarditis on these diseased patients.

Surgical outcomes were excellent, with favorable long-term survival, freedom from reinfection, and HRQOL. These results reinforce the importance of timely aortic valve replacement (AVR) combined with the reconstruction of cardiac anatomy as the benchmark treatment for AVE

## Figures and Tables

**Table 1:** Baseline patient characteristics and risk factors.

Demographic Data	All Patients (*n* = 233/100%)	Native Valve Endocarditis (*n* = 130/55.8%)	Prosthetic Valve Endocarditis (*n* = 103/44.2%)	*p*-Value
Male	200 (85.8%)	111 (85.4%)	89 (86.4%)	0.97
Age, median (IQR), years	66.0 (56.0–73.0)	62.0 (51.0–70.0)	69.8 (62.5–76.2)	<0.05
BMI, median (IQR) (kg/m^2^)	27.1 (24.2–29.9)	26.5 (24.2–29.7)	24.4 (27.7–30.9)	0.16
Comorbidities				
Coronary artery disease	81 (34.8%)	30 (23.1%)	51 (49.5%)	<0.05
PAOD	34 (14.6%)	16 (12.3%)	18 (17.5%)	0.36
COPD	57 (24.5%)	32 (24.6%)	25 (24.3%)	>0.99
Diabetes mellitus	60 (25.8%)	33 (25.4%)	27 (26.2%)	>0.99
Cancer	35 (15.0%)	17 (13.1%)	18 (17.5%)	0.45
Chronic hemodialysis	32 (13.7%)	18 (13.8%)	14 (13.6%)	>0.99
Liver cirrhosis	8 (3.4%)	7 (5.4%)	1 (1.0%)	0.13
NYHA functional class				
I/II	94 (40.3%)	50 (38.5%)	44 (42.7%)	0.60
III/IV	139 (59.7%)	80 (61.5%)	59 (57.3%)	0.60
Intravenous drug abuse	4 (1.7%)	4 (3.1%)	0 (0.0%)	0.13
Embolism	188 (80.7%)	110 (84.6%)	78 (75.7%)	0.12
Cerebral	115 (49.4%)	63 (48.5%)	52 (50.5%)	0.86
Lungs	5 (2.1%)	4 (3.1%)	1 (1.0%)	0.39
Kidneys	18 (7.7%)	12 (9.2%)	6 (5.8%)	0.46
Spleen	47 (20.2%)	29 (22.3%)	17 (16.5%)	0.35
Eyes	4 (1.7%)	3 (2.3%)	1 (1.0%)	0.63
Fever	144 (61.8%)	68 (52.3%)	66 (64.1%)	0.09
Duration of antibiotic therapy (days)	14.6 ± 11.7	13.9 ± 11.9	15.3 ± 11.5	0.42
Logistic EuroSCORE II, %	19.0 ± 14.3%	11.1 ± 8.1%	27.8 ± 14.6%	<0.05
Intubation on mechanical ventilation	22 (9.4%)	12 (9.2%)	10 (9.7%)	>0.99
Cardiac ultrasound findings				
LV-function				
>50%	200 (85.8%)	115 (88.5%)	85 (82.5%)	0.27
40–49%	5 (2.1%)	4 (3.1%)	1 (1.0%)	0.39
30–39%	18 (7.7%)	10 (7.7%)	8 (7.8%)	>0.99
<30%	10 (4.3%)	1 (0.8%)	9 (8.7%)	<0.05
Vegetation present	160 (68.7%)	106 (81.5%)	54 (52.4%)	<0.05
Vegetation Size (mm)	14.1 ± 4.2	13.3 ± 5.2	14.5 ± 6.7	0.27
Abscess present	93 (39.9%)	35 (26.9%)	58 (56.3%)	<0.05

Data presented as median (IQR), mean ± standard deviation or *n* (%). BMI: body mass index.

**Table 2:** Clinical laboratory analyses.

	Native Valve Endocarditis (*n* = 130)	Prosthetic Valve Endocarditis (*n* = 103)	*p*-Value
Serum creatinine (mg/dL)	1.5 ± 1.0	1.5 ± 1.1	0.85
GFR (mL/min/1.73 qm)	64.1 ± 32.3	62.9 ± 28.7	0.78
PCT (ng/mL)	2.6 ± 7.1	5.0 ± 9.7	0.13
CRP (mg/L)	86.3 ± 71.4	88.0 ± 77.6	0.87
WBC (1/nL)	11.1 ± 4.9	11.2 ± 4.1	0.83
NT-proBNP (pg/mL)	17,675 ± 44,002	8160 ± 13,434	0.22

Data presented as mean ± standard deviation or *n* (%).

**Table 3:** Factors predisposing to infective endocarditis.

	All Patients (*n* = 233/100%)	Native Valve Endocarditis (*n* = 130/55.8%)	Prosthetic Valve Endocarditis (*n* = 103/44.2%)	*p*-Value
Dental focus	18 (7.7%)	8 (6.2%)	10 (9.7%)	0.44
Alimentary tract	28 (12.0%)	18 (13.8%)	10 (9.7%)	0.45
Genitourinary tract	3 (1.3%)	3 (2.3%)	0 (0.0%)	0.26
Respiratory tract	9 (3.9%)	6 (4.6%)	3 (2.9%)	0.73
Skin	1 (0.4%)	0 (0.0%)	1 (1.0%)	0.44
Drug addiction	4 (1.7%)	4 (3.1%)	0 (0.0%)	0.13
Trauma	16 (6.9%)	11 (8.5%)	5 (4.9%)	0.41
Chronic hemodialysis	17 (7.3%)	9 (6.9%)	8 (7.8%)	>0.99
Vascular procedures and cardiac catheterization	28 (12.0%)	18 (13.8%)	10 (9.7%)	0.45
Chemotherapy	2 (0.9%)	2 (1.5%)	0 (0.0%)	0.50
ENT	5 (2.1%)	4 (3.1%)	1 (1.0%)	0.39
Meningitis	25 (10.7%)	1 (0.8%)	23 (22.3%)	<0.05
Spondylodiscitis	12 (5.2%)	3 (2.3%)	9 (8.7%)	0.06
Rheumatic heart disease	1 (0.4%)	1 (0.8%)	0 (0.0%)	>0.99
Transplantation immunosuppression	1 (0.4%)	0 (0.0%)	1 (1.0%)	>0.99
No portal of entry apparent	88 (37.8%)	44 (33.8%)	44 (42.7%)	0.21

Data presented as mean ± standard deviation or *n* (%).

**Table 4:** Causal pathogens in 233 cases of infective endocarditis.

Microorganism	All Patients (*n* = 233)	Native Valve Endocarditis (*n* = 130)	Prosthetic Valve Endocarditis (*n* = 103)	*p*-Value
*Staphylococcus aureus*	58 (24.9%)	34 (26.2%)	24 (23.3%)	0.62
*Coagulase-negative staphylococci*	35 (15.0%)	15 (11.5%)	20 (19.4%)	0.06
*Enterococcus faecalis*	29 (12.4%)	16 (12.3%)	14 (13.6%)	0.83
*Enterococcus faecium*	6 (2.6%)	6 (4.6%)	0 (0.0%)	0.06
*Alpha-hemolytic Streptococci (non-Streptococcus pneumoniae)*	20 (8.6%)	18 (13.8%)	2 (1.9%)	<0.05
*Streptococcus mitis group*	15 (6.4%)	12 (9.2%)	3 (2.9%)	0.15
*Streptococcus bovis group*	5 (2.1%)	4 (3.1%)	1 (1.0%)	0.63
*Streptococcus mutans group*	1 (0.4%)	0 (0.0%)	1 (1.0%)	0.46
*Streptococcus salivarius group*	4 (1.7%)	3 (2.3%)	1 (1.0%)	0.63
*Streptococcus anginosus group*	2 (0.9%)	2 (1.5%)	0 (0.0%)	0.51
*Streptococcus agalactiae*	3 (1.3%)	2 (1.5%)	1 (1.0%)	>0.99
*Cutibacterium acnes*	10 (4.3%)	0 (0.0%)	10 (9.7%)	<0.05
*Escherichia coli*	3 (1.3%)	2 (1.5%)	1 (1.0%)	>0.99
*Candida albicans*	2 (0.9%)	1 (0.8%)	1 (1.0%)	>0.99
Negative	40 (17.2%)	15 (11.5%)	24 (23.3%)	0.22

Data presented as mean ± standard deviation or *n* (%).

**Table 5:** Surgical procedures.

	Native Valve Endocarditis (*n* = 130)	Prosthetic Valve Endocarditis (*n* = 103)	*p*-Value
ECC time (min)	95.9 ± 44.0	125.3 ± 56.9	<0.05
Cross-clamp time (min)	83.2 ± 33.7	113.1 ± 58.4	<0.05
Biological valve replacement	96 (73.8%)	88 (85.4%)	<0.05
Mechanical valve replacement	34 (26.2%)	15 (14.6%)	<0.05
Valve reconstruction	1 (0.8%)	0 (0.0%)	>0.99
Concomitant CABG	12 (9.2%)	8 (7.8%)	0.87

Data presented as mean ± standard deviation or *n* (%). CABG: coronary artery bypass grafting, ECC: extracorporeal circulation.

**Table 6:** Postoperative data.

	All Patients (*n* = 233)	Native Valve Endocarditis (*n* = 130)	Prosthetic Valve Endocarditis (*n* = 103)	*p*-Value
In-hospital mortality	31 (13.3%)	11 (8.5%)	19 (18.4%)	<0.05
Reoperation for bleeding	31 (13.3%)	11 (8.5%)	20 (19.4%)	<0.05
Permanent pacemaker implantation	30 (12.9%)	10 (7.7%)	20 (19.4%)	<0.05
Pneumonia	38 (16.3%)	21 (16.2%)	17 (16.5%)	>0.99
Stroke	11 (4.7%)	7 (5.4%)	4 (3.9%)	0.76
Dialysis	30 (12.9%)	10 (7.7%)	20 (19.4%)	<0.05
Low cardiac output with ECLS, *n* (%)	11 (4.7%)	6 (4.6%)	5 (4.9%)	>0.99
ICU stay (days)	6.8 ± 10.1	5.8 ± 6.4	8.0 ± 13.2	0.16
Hospital stay (days)	18.8 ± 17.2	16.1 ± 11.0	22.0 ± 22.1	<0.05
Survival after hospital discharge	173 (74.3%)	98 (75.4%)	75 (72.8%)	0.66
Mortality in follow-up	51 (21.9%)	31 (23.8%)	20 (19.4%)	0.42

Data presented as mean ± standard deviation or *n* (%). ECLS: extracorporeal life support, ICU: intensive care unit. RRT: renal replacement therapy.

**Table 7:** Multivariate logistic regression analysis for in-hospital mortality.

	Odds Ratio (95% CI)	*p*-Value
Aortic cross-clamp time > 76 min	23.1 (2.8–197)	<0.05
Chronic hemodialysis	11.2 (3.5–36.3)	<0.05
*Staphylococcus aureus* infection	4.1 (1.5–11.7)	<0.05
Reduced LV ejection fraction	3.7 (1.1–12.2)	<0.05

## Data Availability

The data that support the findings of the study are available from the corresponding author upon reasonable request.

## Abbreviations

ANOVA	Analysis of variance
AVE	Aortic valve endocarditis
AVR	Aortic valve replacement
CABG	Coronary artery bypass graft
COPD	Chronic obstructive pulmonary disease
CRP	C-reactive protein
ECC	Extracorporal circulation
ECLS	Extracorporeal life support
ENT	Ear, nose, throat
GFR	Glomerular filtration rate
HF	Heart Failure
HRQOL	Health-related quality of life
ICU	Intensive care unit
IE	Infective endocarditis
LV	Left ventricle
LVEF	Left ventricular ejection fraction
MLHFQ	Minnesota Living with HF Questionnaire
MRSA	Methicillin-resistant *Staphylococcus aureus*
NT-proBNP	N-terminal prohormone of brain natriuretic peptide
NVE	Native valve endocarditis
NYHA	New York Heart Association
OR	Odds ratio
PAOD	Peripheral arterial occlusive disease
PCT	Procalcitonin
PVE	Prosthetic valve endocarditis
QOL	Quality of life
RIFLE	Risk, injury, failure, loss of kidney function and end-stage kidney disease
TEE	Transesophageal echocardiography
VSD	Ventricular septum defect
WBC	White blood cell count
